# 979 Missed Appointments, Missed Care: 12-year Review of No-Show/Cancellations in a Burn Clinic

**DOI:** 10.1093/jbcr/iraf019.510

**Published:** 2025-04-01

**Authors:** Kim Priban, Anjay Khandelwal, Tiffany Ciminello

**Affiliations:** Akron Children’s Hospital; Adult and Pediatric Burn Institute at Akron Children’s; Akron Children’s Hospital Burn Center

## Abstract

**Introduction:**

Contemporary trends in burn care have shifted from inpatient to outpatient settings, with APPs and physicians managing more cases. However, data on patient “no-show” and cancellation rates is limited, and the impact of factors like seasonal variations, provider type, and patient demographics remains unclear. This study analyzes 12 years of outpatient burn clinic data to identify trends and factors influencing the visit patterns.

**Methods:**

A 12-year retrospective review of electronic health records was conducted to assess all outpatient clinic visits. The analysis covered APP-led and reconstructive clinics managed by physicians. Key variables included no-show and cancellation rates, reasons for cancellations, appointment day, adults versus pediatrics, and provider type. Data was stratified by year to examine longitudinal trends. Reasons for no-shows or cancellations were recorded either by patients or by clinic staff.

**Results:**

During the study period, 101,595 outpatient visits were scheduled, with an overall no-show rate of 7.3% and a cancellation rate of 14.9%, showing no significant variation over the 12 years. APP-led clinics accounted for 63% of visits, with no-show and cancellation rates of 5.5% and 6.5%, respectively. Physician-led clinics made up 37% of visits and had no-show and cancellation rates of 1.2% and 2.8%. The main reasons for cancellations were patient choice, staff availability, changes in patient condition, and transportation issues, with patient choice and condition changes being slightly more common in APP clinics. No significant differences in cancellation or no-show rates were found by day of the week, season, or between adult and pediatric patients. The pandemic period showed no changes in these trends, and comparison with other outpatient surgical services revealed similar behaviors.

**Conclusions:**

This 12-year analysis of outpatient burn clinic visits provides valuable insights into the trends of no-show and cancellation rates. The findings demonstrate a stable no-show rate and cancellation over the study period, with no significant variations seasonal factors, or patient demographics. Clinics led by advanced practice providers (APPs) accounted for the majority of visits and had slightly higher no-show and cancellation rates compared to those led by physicians. The study highlights common reasons for cancellations, such as patient choice and changes in condition, which were more prevalent in APP-led clinics. These findings align with patterns observed in other outpatient surgical services, indicating consistency in patient behavior across specialties. Further investigation into targeted strategies for reducing no-show and cancellation rates may enhance the efficiency of outpatient burn care services.

**Applicability of Research to Practice:**

Understanding the factors behind outpatient no-show and cancellation rates is crucial for developing strategies to improve clinic efficiency and patient outcomes.

**Funding for the Study:**

N/A

